# Psychometric properties and factor structure of the diabetes eating problem survey – revised (DEPS-R) among adult males and females with type 1 diabetes

**DOI:** 10.1186/s40337-018-0232-0

**Published:** 2019-01-17

**Authors:** Line Wisting, Joseph Wonderlich, Torild Skrivarhaug, Knut Dahl-Jørgensen, Øyvind Rø

**Affiliations:** 1 Regional Department for Eating Disorders, Division of Mental Health and Addiction, Oslo University Hospital, P.O. Box 4956 Nydalen, N-0424 Oslo, Norway; 2 Oslo Diabetes Research Centre, P.O. Box 4956 Nydalen, N-0424 Oslo, Norway; 3 Department of Psychology, George Mason University, Fairfax, VA 22030 USA; 4 Department of Paediatric and Adolescent Medicine, Oslo University Hospital, P.O. Box 4956 Nydalen, N-0424 Oslo, Norway; 5 Institute of Clinical Medicine, Faculty of Medicine, University of Oslo, Problemveien 7, N-0315 Oslo, Norway; 6 The Norwegian Diabetic Centre, Sponhoggveien 19, N-0284 Oslo, Norway; 7 Institute of Clinical Medicine, Mental Health and Addiction, University of Oslo, Problemveien 7, N-0315 Oslo, Norway

**Keywords:** Type 1 diabetes, Eating disorders, Assessment, DEPS-R

## Abstract

**Background:**

Although an increasing amount of research has now established good psychometric properties and a three-component factor structure of the Diabetes Eating Problem Survey – Revised (DEPS-R) in pediatric samples with type 1 diabetes (T1D), research using adult samples has been limited and divergent. This study therefore aimed to investigate psychometric properties and test a three-factor model of the DEPS-R among adults with T1D.

**Methods:**

A total of 282 adults with T1D aged 18–79 years participated in the study. Measures included the DEPS-R, the Eating Disorder Examination Questionnaire (EDE-Q), and clinical data from the Norwegian Quality Improvement of Laboratory Examinations (NOKLUS) system.

**Results:**

The DEPS-R total mean score (SD) for the total sample, males, and females were 13.8 (9.2), 11.2 (7.8), and 15.6 (9.6) respectively. Good fit indices for the confirmatory factor analysis were found. The Cronbach’s alpha of the DEPS-R was .84, suggesting good internal consistency. The DEPS-R correlated significantly with the EDE-Q among both males (.52, *p* < .01) and females (.68, *p* < .001). Also, the DEPS-R correlated significantly with BMI in both genders (.33, *p* < .001 in females and .35, *p* < .001 in males). HbA1c correlated significantly with the DEPS-R in females (.27, *p* < .01), but not in males.

**Conclusions:**

Good fit for a three-factor structure of the DEPS-R was confirmed. Further, the DEPS-R demonstrated good psychometric properties among adults with T1D, and can be recommended for clinical use for this patient group.

## Plain English summary

An increasing amount of research has now established good psychometric properties and a three-component factor structure of the DEPS-R in child and adolescent T1D populations. However, research using adult samples has been limited and divergent. As factor structure may yield relevant clinical information which can guide further treatment approaches, it is important to clarify this analysis. The current study therefore aimed to investigate psychometric properties and test a three-factor model of the DEPS-R among 282 adults with T1D, aged 18–79 years. A confirmatory factor analysis supported the previously reported three factors of the DEPS-R (tapping into maladaptive eating habits, preoccupation with thinness or weight, and the concept of maintaining high blood glucose values to lose weight) among an adult type 1 diabetes population. Furthermore, this study reports good internal consistency and adequate construct validity, as the DEPS-R correlated significantly with the EDE-Q among males and females. In conclusion, the previously reported three factor structure of the DEPS-R was supported in the current study. Further, the DEPS-R demonstrated overall good psychometric properties among adults with T1D and can be recommended for clinical use for this patient group.

## Background

Disordered eating and eating disorders are commonly reported in T1D, with a 2–3 fold increase in prevalence of eating disorders compared to individuals without T1D according to meta-analyses [1–3]. Disordered eating, and especially behavior leading to incorrect insulin dosing, is a major hindrance to optimal blood glucose control which is necessary to prevent severe late diabetes complications, and increased mortality. Prevalence of disordered eating varies largely across individual studies, most likely due to assessment differences. Specifically, generic eating disorder measures made for the general population, including the Eating Attitudes Test (EAT) [4], the SCOFF (acronym created from the letters of the items, i.e. *do you make yourself* ***S****ick because you feel uncomfortably full; do you worry you have lost* ***C****ontrol over how much you eat; have you recently lost more than* ***O****ne stone in a 3 month period; do you believe yourself to be* ***F****at when others say you are too thin*; *and would you say that* ***F****ood dominates your life*) [5], and the Eating Disorder Examination – Questionnaire (EDE-Q) [6], have been reported to yield elevated prevalence estimates among patients with T1D compared to diabetes-specific tools [3]. This may be related to the dietary monitoring naturally occurring as part of standard T1D treatment being scored pathologically, implying a risk of false positives. Additionally, generic assessment tools do not account for intentional insulin under dosing or omission to control weight, a diabetes-specific compensatory behavior reported in up to 37% of females with T1D [7–9] and associated with a three-fold mortality rate [7]. As such, diabetes-specific measures to assess disordered eating are recommended in diabetes populations [3].

The Diabetes Eating Problem Survey – Revised (DEPS-R) [10] was the first screening tool for disordered eating designed specifically for diabetes and its psychometric properties among children and adolescents have been established in English [10], German [11], Turkish [12], and Norwegian [13]. The Norwegian child and adolescent study [13] also explored the factor structure of the DEPS-R, yielding three factors tapping into maladaptive eating habits, preoccupation with thinness or weight, and maintaining high blood glucose values to lose weight. Although the psychometric properties (e.g. internal consistency, construct validity, convergent validity, external validity, and criterion validity) are established among children and adolescents, only two studies have validated the DEPS-R in a T1D sample that included adults [14, 15], confirming satisfactory psychometric properties in terms of internal consistency (Cronbach’s alpha typically reported at .80 and above) and construct validity. However, the two adult validation studies are inconsistent in terms of factor structure. Sancanuto et al. [15] conducted an exploratory factor analysis in 112 adults with T1D, yielding five factors (eating attitudes, bulimic behavior, weight control, avoidance, and restriction). In contrast, Pinna et al. [14] conducted a confirmatory factor analysis of the DEPS-R in a sample of patients with insulin-treated diabetes (192 with T1D), fully conforming to those reported among Norwegian adolescents [13]. However, they used fit indices that have since been revised to be more conservative, which might not reflect the best fitting model. Recent research has suggested that more conservative fit indices should be used when using structural equation modelling (e.g. the criteria for CFI has been changed from .9 to .95 for good fit) [16]. Also, the sample was mixed between T1D and type 2 diabetes, and between adolescents and adults. The three-factor solution needs to be examined with a T1D adult sample only. This is relevant not only for scientific purposes, but also for clinicians when adopting this measure with patients in their clinics. Finally, the psychometric properties of the Norwegian version of the DEPS-R among adults have not previously been examined.

This study therefore aimed to investigate the psychometric properties and factor structure of the DEPS-R (Norwegian version) in adult males and females with T1D. The study also has the largest sample to date of the existing DEPS-R validation studies among adults, and it is the first study among adults to validate the DEPS-R against the EDE-Q.

## Methods

### Design

This is a cross-sectional design study.

### Participants and procedure

Patients with T1D were recruited from the Norwegian Diabetic Centre (NDC) between February 2016 and October 2017. The NDC is an outpatient clinic for adults (approximately 1300) with T1D, located in Oslo. Patients from Oslo and surrounding areas are referred to the NDC by both general practitioners and hospitals. The NDC is a multidisciplinary clinic organized under the Norwegian Health South-East Authority. Questionnaires were completed as part of a routine diabetes consultation at the outpatient clinic. Data for this paper was collected as part of a larger study, and the current sample is the same as reported in a previous article [17]: a total of 282 males and females (60% females) aged 18–79 years (mean age 42.1; SD: 15.2). Mean age of T1D onset was 15.1 years (SD: 11.2), mean HbA1c was 7.8% (62 mmol/mol) (SD: .9), mean T1D duration was 27.1 years (SD: 14.4), and mean body mass index (BMI) was 26.0 (SD: 4.1). A total of 56.3% administered insulin with an insulin pen and 43.3% with a pump. Table 1 illustrates patient characteristics. The regional ethics committee approved the study, and written consent was obtained from all participants.

**Table 1:** Participant characteristics

	All	Males	Females	Sig. level
	*N* = 282	*N* = 112 (40%)	*N* = 170 (60%)	
Age (years)	42.1 (15.2)	44.6 (15.9)	40.5 (14.5)	.05
Diabetes onset (age)	15.1 (11.2)	15.4 (10.9)	14.9 (11.4)	ns
HbA1c	7.8% (62 mmol/mol)	7.6% (60 mmol/mol)	7.9% (63 mmol/mol)	ns
	(.9)	(.9)	(.9)	
Diabetes duration (years)	27.0 (14.4)	29.1 (14.8)	25.7 (14.1)	ns
BMI self-report	26.0 (4.1)	26.5 (3.8)	25.6 (4.3)	ns
Mode of insulin treatment	56.3% pen	60.9% pen	53.4% pen	
	43.3% pump	38.0% pump	46.6% pump	

Data are means (SD) and significance level. HbA1c is reported as % and millimoles per mole (mmol/mol). BMI is expressed in units of kg/m^2^

### Measures

The Diabetes Eating Problem Survey – Revised (DEPS-R) [10] is a diabetes-specific screening tool for disordered eating and consists of 16 items, derived from the 28-item Diabetes Eating Problem Survey (DEPS) [18]. Responses are scored on a 6-point Likert scale and higher scores indicate greater eating disorder pathology. The predetermined cut-off score for disordered eating is empirically established at 20 or above [10], indicating individuals with a level of disordered eating warranting further attention. The DEPS-R has been translated to Norwegian and validated in an adolescent sample aged 11–19 years, demonstrating good psychometric properties [13]. The DEPS-R is typically completed in less than 10 min.

The Eating Disorder Examination Questionnaire (EDE-Q) [6] is a self-report questionnaire of specific eating disorder psychopathology based on the Eating Disorder Examination (EDE) diagnostic interview [19]. It consists of the four subscales *eating restraint*, *eating concern*, *shape concern*, and *weight concern*. As the literature provides mixed support for these subscales [20–22], the present study reports the overall global score only [20]. The EDE-Q is previously translated and validated in Norwegian males and females [23, 24]. Weight and height are self-reported in the EDE-Q. BMI was calculated based on self-reported weight and height (kg/m^2^) from the EDE-Q.

Clinical data was assessed via the Norwegian Quality Improvement of Laboratory Examinations (NOKLUS) system [25], and was conducted as part of standard clinical assessment at the NDC. T1D clinical data include HbA1c, T1D onset, and treatment mode. HbA1c is a measure of long term blood glucose levels and reflects average blood glucose the preceding 8–12 weeks. HbA1c is used here as a measure of metabolic control. A reasonable HbA1c target for many nonpregnant adults is < 7.0% (53 mmol/mol). The providers might reasonably suggest a more stringent HbA1c goal such as 6.5% (48 mmol/mol) for selected individual patients if this can be achieved without significant hypoglycemia or other adverse effects of treatment [26].

### Data analyses

Construct validity was investigated by means of Pearson correlation to explore relationships between the DEPS-R and EDE-Q total scores. Furthermore, correlation analyses were also carried out to investigate the association between the DEPS-R and other constructs hypothesized to covary with DEPS-R scores, such as age and BMI. In line with Cohen [27], correlations of .10 to .29 were interpreted as small, .30 to .49 as medium and .50 to 1.0 as large. Internal consistency was assessed by Cronbach’s alpha coefficients. Independent samples t-tests were carried out to investigate group differences. Alpha level was set to *p* < .05. Effect sizes were calculated by means of Cohen’s *d*. Following the guidelines by Cohen [27], effect sizes > .2 were interpreted as small, > .5 as medium and > .8 as large. The three-factor model described previously [13, 14] was tested using confirmatory factor analysis with the maximum likelihood discrepancy method. Additionally, a one-factor and a five-factor model were tested. Fit indexes indicate a good fit when: χ^2^ (CMIN) is non-significant (*p* > .05); HOELTER.05 > 200; Standardized Root Mean Square Residual (SRMR) < .08; Root Mean Square Error of Approximation (RMSEA) < .05 (good fit) and .05 to .1 (moderate fit); PCLOSE > .05; Comparative Fit Index (CFI) > .95 (good fit), > .90 (traditional fit), > .8 (sometimes permissible) [28]; Normed Fit Index (NFI) > .95; NNFI (TLI) > .991; Akaike Information Criterion (AIC) (smaller values indicate a better model fit) [12, 16, 28]. Six variables had missing values, all less than 5% missing, which were replaced with the median values given the variables assess on an ordinal scale. Modification indexes were examined to determine the co-variance between errors that improved the model fit. Specifically, criteria for covariant errors followed suggestions delineated by Kenny (e.g. large modification indices > 15) [29]. Statistical analyses were conducted using SPSS version 23 (SPSS IBM, NY, USA) [30]. The confirmatory factor analysis was conducted using IBM® SPSS® Amos™ 20.0.

## Results

**Fig. 1:**
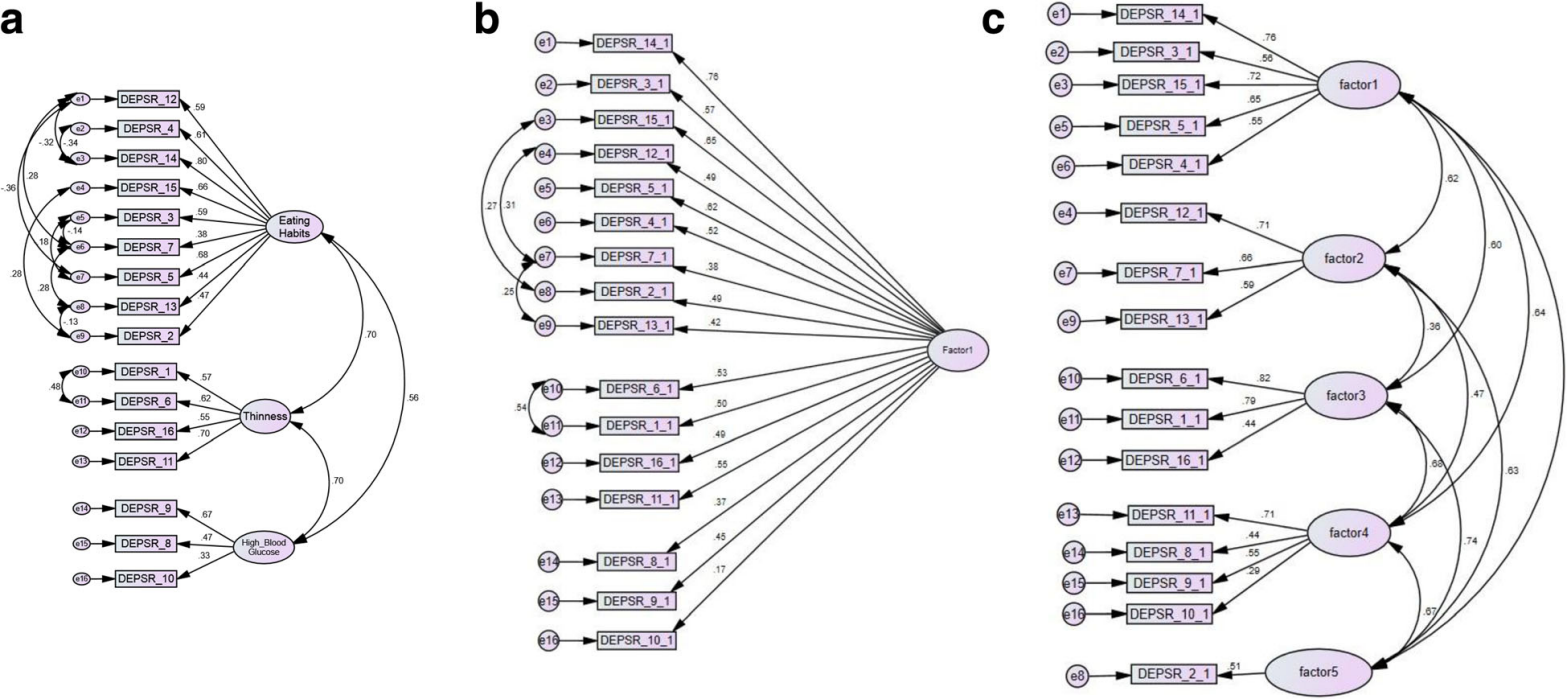
**a** Confirmatory factor analysis of the three-factor model. CMIN = 158.55; DF = 91; *p =* 0.000; CMIN/df = 1.74; HOELTER.05 = 132; SRMR = 0.05; RMSEA = 0.05; PCLOSE = 0.42; CFI = 0.95; NFI = 0.884; TLI = 0.947; AIC = 244.55. **b** One-factor model (covaried errors with modification indices above 15). CMIN = 284.55; DF = 100; *p* = 0.000; CMIN/df = 2.84; HOELTER.05 = 123; SRMR = 0.087; RMSEA = 0.091; PCLOSE = 0.00; CFI = 0.825; NFI = 0.792; TLI = 0.822; AIC = 365.79. **c** Five-factor model (no modification indices above 15 thus no covaried errors). CMIN = 245.49; DF = 95; *p =* 0.000; CMIN/df = 2.697; HOELTER.05 = 132; SRMR = 0.06;RMSEA = 0.086; PCLOSE = 0.00; CFI = 0.872; NFI = 0.814; TLI = 0.832; AIC = 365.79

As shown in Fig. 1a, 1b, and 1c, we compared a one-factor, a three-factor, and a five-factor model. Given the models were not nested, we cannot provide direct comparisons (e.g. delta chi square), however as seen in Tables 2, 3 and 4 the three-factor model provided the most good fit indices. The three factors (maladaptive eating habits, preoccupation with thinness or weight, and maintaining high blood glucose to lose weight) were further correlated with HbA1c and the DEPS-R total score, with significant associations between HbA1c with the first (.24, *p* < .001) and third factor (.14, *p* < .05). Factor 2 was not significantly correlated with HbA1c (.10, ns). The DEPS-R total score correlated significantly with all three factors, with correlation coefficients of .91 (*p* < .001), .80 (*p* < .001), and .50 (*p* < .001), respectively. The Cronbach’s alpha of the DEPS-R was .84, suggesting good internal consistency. When split by gender, the Cronbach’s alpha was .84 for females and .81 for males. Internal consistency was also explored for the three factors identified in the confirmatory factor analysis, yielding Cronbach’s alpha of .80 for factor 1, .74 for factor 2, and .48 for factor 3. The Cronbach’s alpha of the EDE-Q global score for the total sample, males, and females, was .94, .92, and .94, respectively. As described in Table 2, the DEPS-R correlated significantly with the EDE-Q, with large correlations among both males (.52, *p* < .01) and females (.68, *p* < .001). Also, the DEPS-R correlated significantly with BMI in both genders, with medium-sized correlations of .33 (*p* < .001) in females and .35 (*p* < .001) in males. Furthermore, HbA1c was significantly (although small-sized) correlated with the DEPS-R in females (.27, *p* < .01), but not in males. As for descriptive data, and as previously reported [17], the DEPS-R total mean score (SD) for the total sample, males, and females were 13.83 (9.2), 11.18 (7.8), and 15.57 (9.6) respectively.

**Table 2:** Description of various fit indices for the different factor models with and without covaried errors based on modification indices larger than 15

	1-Factor Model		3-Factor Model		5-factor Model^a^
	Covaried errors	No covaried errors	Covaried errors	No covaried errors	No covaried errors
CMIN	284.55	447.63	158.55	330.64	245.49
df	100	104	91	`101	95
*p*-value of CMIN	0.000	0.000	0.000	0.000	0.000
CMIN/df	2.845^b^	4.304^b^	1.741 ^b^	3.271 ^b^	2.584 ^b^
SRMR	0.087	0.817	0.050 ^b^	0.0734 ^b^	.079 ^b^
RMSEA	0.091	.108	0.050^c^	0.080 ^b^	0.086
PCLOSE	0.000	0.000	0.420 ^b^	0.000	0.000
CFI	0.825	0.724	0.95 ^c^	0.88	0.872
TLI	0.822	0.682	0.947 ^b^	0.785	0.832
NFI	0.792	0.673	0.884	0.822	0.814
AIC	365.79	511.631	244.55	400.364	365.79

^a^No data provided for the five-factor model with covaried errors because the five-factor model did not have any modification indices above 15

^b^indicates good fit

^c^indicates great fit

**Table 3:** Construct validity of the DEPS-R in males and females, as indicated by the correlation between the DEPS-R total score and EDE-Q global score, as well as with HbA1c, BMI, and age

	All	Males	Females
EDE-Q	.67^a^	.52 ^b^	.68 ^a^
HbA1c	.22^b^	ns	.27 ^b^
BMI	.31 ^a^	.35 ^a^	.33 ^a^
Age	−.27 ^a^	ns	−.32 ^a^

^a^= alpha level .001

^b^= alpha level .01; *ns* not significant

**Table 4:** Correlations between the three DEPS-R factors and the EDE-Q global score, HbA1c, BMI, and age for the total sample (males and females; *N =* 282)

	Factor 1	Factor 2	Factor 3
EDE-Q	.54***	.67***	.43***
HbA1c	.24***	ns	.14*
BMI	.18**	.39***	.19**
Age	−.30***	−.14*	ns

***= alpha level .001; **= alpha level .01; *= alpha level .05; *ns* not significant

## Discussion

This study reported adequate internal consistency and construct validity of the Norwegian translation of the DEPS-R among adults with T1D. This is generally consistent with existing literature on DEPS-R among children and adolescents [10, 11, 13, 31] as well as the two studies among adults with T1D [14, 15]. This was the first study to establish construct validity with the EDE-Q, a widely used and by many the preferred measure of specific eating disorder psychopathology. Significant and large correlations (.52 (*p* < .01) in males and .68 (*p* < .001) in females) between the DEPS-R and EDE-Q suggest good construct validity of the DEPS-R among adults with T1D.

Furthermore, a confirmatory factor analysis supported the previously reported three-factor solution describing maladaptive eating, preoccupation with thinness, and the concept of maintaining high blood glucose levels to lose weight. This is in full concordance with our previous study among children and adolescents [13] and the Italian study by Pinna et al. [14], however now confirmed in a purely T1D adult population. We acknowledge that Sancunato et al. [15] in their exploratory factor analysis of the DEPS-R identified five factors in their Spanish adult validation study. However, one of their factors had only one item loading on it, which is not recommended for a confirmatory factor analysis [26]. Although the existing one-factor structure is recommended in clinical practice for efficient screening for disordered eating, a more detailed description of the factor structure is important to establish for descriptive purposes, as well as to get a broader sense of the nature of the eating disorder psychopathology and to tailor the subsequent treatment to individual needs. For example, a patient scoring higher on factor 3 (concept of maintaining high blood glucose values to lose weight) may benefit from a different treatment approach than patients scoring low on factor 3, but high on factor 1 (maladaptive eating) or 2 (preoccupation with thinness). To further evaluate the reported three-factor solution, the factors were correlated with the DEPS-R total score and HbA1c. All three factors correlated relatively strongly with the DEPS-R total score, with correlation coefficients ranging from .50 to .91. With regard to metabolic control, a construct previously reported to be associated with eating disorder psychopathology and associated with the onset of serious late diabetes complications, factors one and three were significantly associated with HbA1c, whereas factor 2 was not. These associations were generally similar to those of the Norwegian pediatric sample, suggesting similar factor structure across these age groups. Furthermore, factor one was more strongly correlated with both the DEPS-R total score and with HbA1c than factors two and three in both studies, suggesting factor one to be most dominant factor in the reported three-factor structure. Finally, factor three scored lower than factor one and two in terms of both Cronbach’s alpha (.48 compared to .80 and .74) and correlation with the DEPS-R total score (.50 compared to .91 and .80). This may be a reflection of factor three having the fewest number of items loading on it, but nevertheless may suggest that the scientific support for factor one and two is stronger, which should be noted when interpreting results from factor three.

DEPS-R mean scores and standard deviations were reported for the total sample, males, and females in the present study, yielding scores of 13.8 (9.2), 11.2 (7.8), and 15.6 (9.6), respectively. Such descriptive data have also been reported in previous DEPS-R validation studies in adults. Sancanuto et al. [15] reported mean scores of 6.8 (SD 6.6) for males and 16.5 (SD 7.7) for females, whereas Pinna et al. [14] reported a median score of 12 in the total population, 10 in males and 14 in females. These adult mean scores are generally comparable to those of pediatric and adolescent samples [10, 11, 13, 31]. Thus, mean scores in females are generally comparable across studies, with more variation among males. The observed gender differences in levels of eating disorder psychopathology across all DEPS-R studies are consistent with existing literature, both in diabetes [3, 9, 32] and non-diabetes samples [33]. In fact, it has been suggested that eating disorder measures may represent a gender bias since they are developed and validated within female samples [34]. Whereas the eating disorder psychopathology among females is typically characterized by pursue of the thin body ideal, males may be more concerned with muscularity [35, 36].

The current study has the largest sample to date of the existing DEPS-R validation studies among adults, which represents a strength of the study. Also, it is the first study among adults to validate the DEPS-R against the EDE-Q, derived from the EDE diagnostic interview, and by many described as the preferred measure of specific eating disorder psychopathology. Finally, the inclusion of males and older adults represents strengths of the current study as many previous studies include young females only. However, the study is limited by its cross-sectional design and is lacking a test-retest reliability assessment. Furthermore, although this is the largest sample among the DEPS-R adult validation studies, the sample size is still modest. Data is collected from one clinic only, thus we cannot be certain about the generalizability of the findings. Finally, we did not include a clinical interview which could have provided diagnostic information. Future studies should aim to validate the DEPS-R against a diagnostic interview conducted by a professional.

## Conclusions

A valid screening tool for disordered eating is potentially an important addition to clinical practice. Given the frequency and severity of comorbid T1D and disordered eating, early detection and subsequent intervention is crucial to minimize the risk of serious diabetes complications. The short time required to complete, score, and interpret the instrument is crucial in a busy clinical setting with little available time. In conclusion, the Norwegian translation of the DEPS-R has adequate psychometric properties and can be recommended for clinical use, both in adolescent and adult populations (11 years and above).

## Abbreviations

BMI: Body mass index
DEPS: Diabetes eating problem survey
DEPS-R: The diabetes eating problem survey – revised
EAT: Eating attitudes test
EDE: Eating disorder examination
EDE-Q: Eating disorder examination questionnaire
HbA1c: Hemoglobin A1c
NDC: The Norwegian diabetic center
NOKLUS: The Norwegian quality improvement of laboratory examination
SPSS: The statistical package for the social sciences
T1D: Type 1 diabetes

## References

[1] Mannucci E, Rotella F, Ricca V, Moretti S, Placidi GF, Rotella CM (2005). Eating disorders in patients with type 1 diabetes: a meta-analysis. JEndocrinolInvest.

[2] Nielsen S (2002). Eating disorders in females with type 1 diabetes: an update of a meta-analysis. Eur Eat Disord Rev.

[3] Young V, Eiser C, Johnson B, Brierley S, Epton T, Elliott J, et al. Eating problems in adolescents with Type 1 diabetes: a systematic review with meta-analysis. Diabet Med. 2013;30(2):189-98. 10.1111/j.1464-5491.2012.03771.x 10.1111/j.1464-5491.2012.03771.x 22913589

[4] Garner DM, Olmsted MP, Bohr Y, Garfinkel PE (1982). The eating attitudes test: psychometric features and clinical correlates. PsycholMed.

[5] Morgan JF, Reid F, Lacey JH (1999). The SCOFF questionnaire: assessment of a new screening tool for eating disorders. BMJ.

[6] Fairburn CG, Beglin SJ (1994). Assessment of eating disorders: interview or self-report questionnaire?. IntJEatDisord.

[7] Goebel-Fabbri AE, Fikkan J, Franko DL, Pearson K, Anderson BJ, Weinger K (2008). Insulin restriction and associated morbidity and mortality in women with type 1 diabetes. Diabetes Care.

[8] Peveler RC, Bryden KS, Neil HA, Fairburn CG, Mayou RA, Dunger DB (2005). The relationship of disordered eating habits and attitudes to clinical outcomes in young adult females with type 1 diabetes. Diabetes Care.

[9] Wisting L, Froisland DH, Skrivarhaug T, Dahl-Jorgensen K, Ro O (2013). Disturbed eating behavior and omission of insulin in adolescents receiving intensified insulin treatment: a nationwide population-based study. Diabetes Care.

[10] Markowitz JT, Butler DA, Volkening LK, Antisdel JE, Anderson BJ, Laffel LM (2010). Brief screening tool for disordered eating in diabetes: internal consistency and external validity in a contemporary sample of pediatric patients with type 1 diabetes. Diabetes Care.

[11] Sassmann H, Albrecht C, Busse-Widmann P, Hevelke LK, Kranz J, Markowitz JT (2015). Psychometric properties of the German version of the diabetes eating problem survey-revised: additional benefit of disease-specific screening in adolescents with type 1 diabetes. Diabetic medicine : a journal of the British Diabetic Association.

[12] Atik Altinok Y, Ozgur S, Meseri R, Ozen S, Darcan S, Goksen D (2017). Reliability and validity of the diabetes eating problem survey in Turkish children and adolescents with type 1 diabetes mellitus. Journal of clinical research in pediatric endocrinology.

[13] Wisting L, Froisland DH, Skrivarhaug T, Dahl-Jorgensen K, Ro O (2013). Psychometric properties, norms, and factor structure of the diabetes eating problem survey-revised in a large sample of children and adolescents with type 1 diabetes. Diabetes Care.

[14] Pinna F, Diana E, Sanna L, Deiana V, Manchia M, Nicotra E (2017). Assessment of eating disorders with the diabetes eating problems survey - revised (DEPS-R) in a representative sample of insulin-treated diabetic patients: a validation study in Italy. BMC psychiatry.

[15] Sancanuto C, Jimenez-Rodriguez D, Tebar FJ, Hernandez-Morante JJ (2017). Translation and validation of the diabetes eating problem survey to screen eating disorders in patients with type-1 diabetes mellitus. Medicina clinica.

[16] Hair JF, Black WC, Babin BJ, Anderson RE. Multivariate data analysis: A global perspective. New Jersey: Pearson Prentice Hall; 2010.

[17] Wisting L, Skrivarhaug T, Dahl-Jorgensen K, Ro O (2018). Prevalence of disturbed eating behavior and associated symptoms of anxiety and depression among adult males and females with type 1 diabetes. J Eat Disord.

[18] Antisdel JE, Laffel LMB, Anderson BJ. Improved detection of eating problems in women with type 1 diabetes using a newly developed survey. Diabetes. 2001;50(Suppl. 1):A47.

[19] Fairburn C, Cooper Z, Fairburn C, Wilson G (1993). The eating disorder examination. Binge eating: nature, assessment, and treatment.

[20] Byrne SM, Allen KL, Lampard AM, Dove ER, Fursland A (2010). The factor structure of the eating disorder examination in clinical and community samples. The International journal of eating disorders.

[21] Grilo CM, Reas DL, Hopwood CJ, Crosby RD (2015). Factor structure and construct validity of the eating disorder examination-questionnaire in college students: further support for a modified brief version. The International journal of eating disorders..

[22] White HJ, Haycraft E, Goodwin H, Meyer C (2014). Eating disorder examination questionnaire: factor structure for adolescent girls and boys. The International journal of eating disorders..

[23] Reas DL, Øverås M, Rø Ø. Norms for the eating disorder examination questionnaire (EDE-Q) among high school and university men. Eating disorders: The journal of treatment and Prevention. 2012;20(5):437-43. 10.1080/10640266.2012.715523. 10.1080/10640266.2012.715523 22985240

[24] Ro O, Reas DL, Lask B (2010). Norms for the eating disorder examination questionnaire among female university students in Norway. Nordic journal of psychiatry.

[25] Norwegian Quality Improvement of Laboratory Examinations. 2018. Available from: http://www.noklus.no/en/Home.aspx. Accessed 20 Dec 2018.

[26] StatisticsSolutions. Confirmatory Factor Analysis 2013 [Available from: http://www.statisticssolutions.com/confirmatory-factor-analysis/. Accessed 20 Dec 2018.

[27] Cohen J (1988). Statistical power analysis for the Behavioural sciences.

[28] Lt H, Bentler PM (1999). Cutoff criteria for fit indexes in covariance structure analysis: conventional criteria versus new alternatives. Struct Equ Model Multidiscip J.

[29] Kenny D (2011). Respecification of Latent Variable Models.

[30] Corp. I (2015). IBM SPSS Statistics for Windows, Version 23.

[31] Atik Altinok Y, Ozgur S, Meseri R, Ozen S, Darcan S, Goksen D. RELIABILITY AND VALIDITY OF THE DIABETES EATING PROBLEM SURVEY-REVISED ON TURKISH CHILDREN AND ADOLESCENTS WITH TYPE 1 DIABETES MELLITUS. Journal of clinical research in pediatric endocrinology. 2017;9(4):323–8. 10.4274/jcrpe.4219 PMC5785638 28270369

[32] Wisting L, Bang L, Skrivarhaug T, Dahl-Jorgensen K, Ro O (2015). Adolescents with type 1 diabetes - the impact of gender, age, and health-related functioning on eating disorder psychopathology. PLoS One.

[33] Lindvall Dahlgren C, Wisting L, Ro O (2017). Feeding and eating disorders in the DSM-5 era: a systematic review of prevalence rates in non-clinical male and female samples. J Eat Disord.

[34] Lindvall Dahlgren C, Wisting L (2016). Transitioning from DSM-IV to DSM-5: a systematic review of eating disorder prevalence assessment. The International journal of eating disorders..

[35] Mayo C, George V (2014). Eating disorder risk and body dissatisfaction based on muscularity and body fat in male university students. Journal of American college health : J of ACH.

[36] Mitchison D, Mond J (2015). Epidemiology of eating disorders, eating disordered behaviour, and body image disturbance in males: a narrative review. J Eat Disord.

